# The Role of Cardiovascular Magnetic Resonance Imaging in the Assessment of Mitral Regurgitation

**DOI:** 10.3390/diagnostics14060644

**Published:** 2024-03-19

**Authors:** Ioannis Botis, Maria-Anna Bazmpani, Stylianos Daios, Antonios Ziakas, Vasileios Kamperidis, Theodoros D. Karamitsos

**Affiliations:** First Department of Cardiology, AHEPA Hospital, Aristotle University of Thessaloniki, 54636 Thessaloniki, Greece; jbotis@hotmail.com (I.B.); stylianoschrys.daios@gmail.com (S.D.); vkamperidis@outlook.com (V.K.)

**Keywords:** cardiovascular magnetic resonance, mitral regurgitation, review, quantification, parametric mapping, late gadolinium enhancement, feature tracking

## Abstract

Mitral regurgitation (MR), a primary cause of valvular disease in adults, affects millions and is growing due to an ageing population. Cardiovascular magnetic resonance (CMR) has emerged as an essential tool, offering insights into valvular and myocardial pathology when compared to the primary imaging modality, echocardiography. This review highlights CMR’s superiority in high-resolution volumetric assessment and tissue characterization, including also advanced techniques like late gadolinium enhancement imaging, parametric mapping, feature tracking and 4D flow analysis. These techniques provide a deeper understanding of MR’s pathophysiology and its effect on cardiac chambers, enabling CMR to surpass echocardiography in predicting hard clinical outcomes and left ventricular (LV) remodelling post mitral valve surgery. Despite its advantages, CMR’s application faces limitations like cost, lack of standardization, and susceptibility to arrhythmia artifacts. Nonetheless, as technological advancements continue and new evidence emerges, CMR’s role in MR assessment is set to expand, offering a more nuanced and personalized approach to cardiac care. This review emphasizes the need for further research and standardized protocols to maximize CMR’s potential in MR management.

## 1. Introduction

Mitral regurgitation (MR) emerges as the most common valvular heart disease worldwide affecting 1–2% of the global population and more than two million patients in the USA alone [1,2]. Its prevalence increases from less than 1% in individuals younger than 55 to almost 10% in people aged 75 and above [1]. MR can be either primary or secondary, and its aetiology varies globally [2]. In developing countries, rheumatic heart disease accounts for the vast majority of primary MR cases, while in developed countries primary MR most often results from degeneration of the valve or the subvalvular apparatus [2,3]. Secondary MR is predominantly caused by ischaemic heart disease or cardiomyopathy [2,3].

Cardiac imaging is essential for the identification and the grading of the severity of MR. Even though echocardiography remains the first-line imaging modality for the assessment of MR, cardiovascular magnetic resonance (CMR) is increasingly recognized as an alternative diagnostic tool [4]. Real-world evidence has already made CMR the reference standard for the precise quantification of atrial and ventricular volumes and function, utilizing the widely available balanced Steady State Free Precession (SSFP) sequences and the summation of the short-axis slices of cavities, without the use of any geometrical assumptions [5,6]. CMR boasts several advantages, as it can provide unrestricted imaging planes of the whole heart, without ionizing radiation or the need for iodinated contrast agents. In that direction, both 2021 ESC/EACTS and 2020 ACC/AHA valvular heart disease guidelines recommend CMR as an alternative quantification tool, especially in cases where MR severity assessment is inconclusive due to very eccentric jets, suboptimal echocardiographic image quality, discrepancies among echocardiographic indices or a mismatch between clinical symptoms and imaging findings [7,8]. Lastly, beyond mere quantification, techniques like late gadolinium enhancement, parametric mapping, feature tracking, and 4D flow analysis, delve deeper, revealing subtle changes in the myocardial tissue invisible using other methods and providing a more comprehensive evaluation of the aetiology and the consequences of mitral valve disease.

The aim of this review is to provide a practical guide on how to comprehensively assess with CMR the mitral valve and the left ventricle in patients with mitral regurgitation.

## 2. How to Assess Mitral Regurgitation with CMR

**Figure 1:**
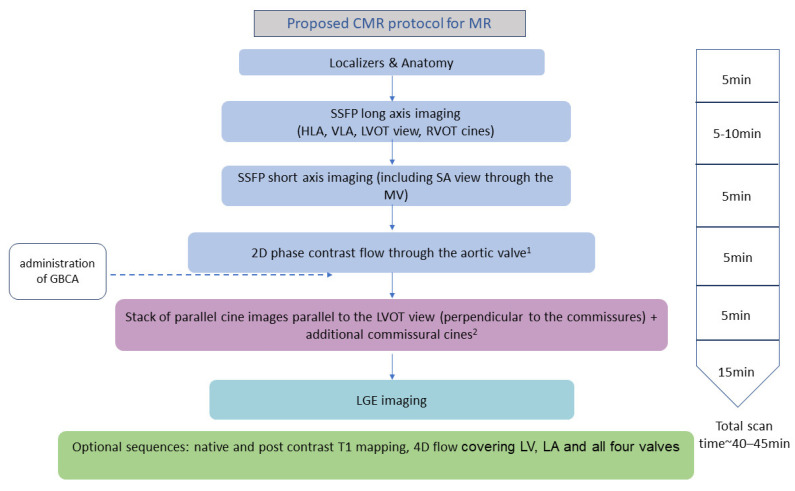
Proposed CMR protocol for the assessment of mitral valve regurgitation. CMR, cardiovascular magnetic resonance; MR, mitral regurgitation; HLA, horizontal long axis; VLA, vertical long axis; LVOT, left ventricular outflow tract; SA, short axis; GBCA, gadolinium based contrast agent; LGE, late gadolinium enhancement; LV, left ventricle; LA, left atrium. 1. Not necessary if 4D flow performed. 2. Can be obtained before or immediately after administration of GBCA.

**Figure 2:**
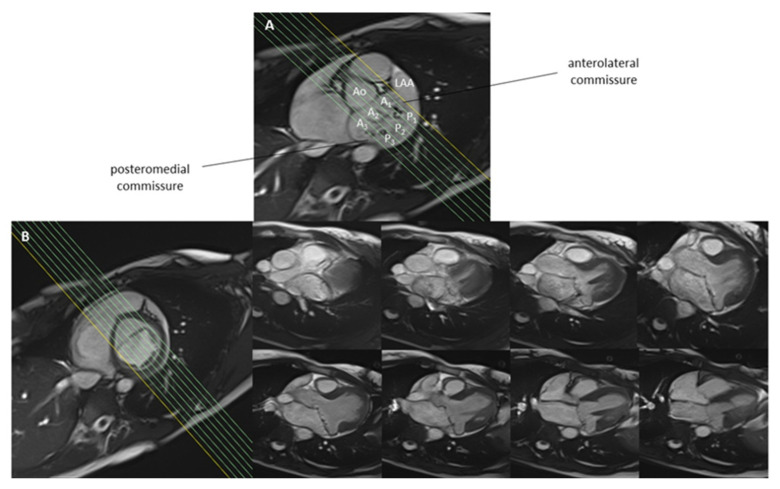
Panel (**A**). Short axis diagram of the mitral valve showing all scallops of mitral valve leaflets (A_1_-P_1_, A_2_-P_2_, A_3_-P_3_) in a patient with Barlow’s disease. Panel (**B**). A stack of parallel cine images obtained parallel to left ventricular outflow tract (perpendicular to the commissure) allows a systematic assessment of all the mitral valve cusps. LAA, left atrial appendage; Ao, aortic valve.

A detailed CMR assessment of the mitral valve should begin with the basic protocol recommended by the Society of Cardiovascular Magnetic Resonance (SCMR) [9] and also include details about the morphological and functional characteristics of the mitral valve leaflets, chordae tendineae, and annulus. A suggested CMR scanning protocol is shown in Figure 1. While echocardiography, as a primary diagnostic tool, often provides detailed insights into the regurgitation mechanism, CMR, despite its lower temporal resolution, can still provide valuable information about the dynamic behaviour of each component of the mitral valve apparatus [10]. This includes each scallop, which can be visualised if additional contiguous modified three-chamber cines intersecting the commissural line are planned, as visualised in Figure 2 [11]. This approach is often sufficient to localize segmental pathologies such as billowing, prolapse, flail, thickening, or calcification [12,13]. CMR cines can also possibly detect mitral annulus disjunction, which is an abnormal atrial displacement of the mitral valve leaflet hinge point, often associated with mitral valve prolapse (MVP) [14,15,16]. CMR can accurately measure the extent of the detachment of the mitral annulus from the ventricular myocardium [14,16,17].

MR jet characteristics, such as jet eccentricity, the number of jets and their duration during systole should also be described. Cine acquisitions facilitate this, utilizing spin–spin dephasing from flow turbulence, which creates hypointense areas in the blood pool, thus aiding in qualitative MR assessment. However, there is an inherent limitation of the SSFP images in visualising flow dynamics; they are highly susceptible to significant variations of the specific signal loss area with minor alterations in sequence parameters [18]. This variability negatively affects the sensitivity of the technique which is why visual assessment of the jet is generally not recommended for MR severity estimation and is only used for crude information regarding its location and direction [18]. Alternatively, Fast Spoiled Gradient echo sequences with longer repetition and echo times can be more sensitive to depicting flow changes and MR regurgitant flow voids [6]. Lastly, incorporating information about MR aetiology, and using Carpentier’s classification system in the CMR report could be useful in guiding management and the type of intervention.

**Figure 3:**
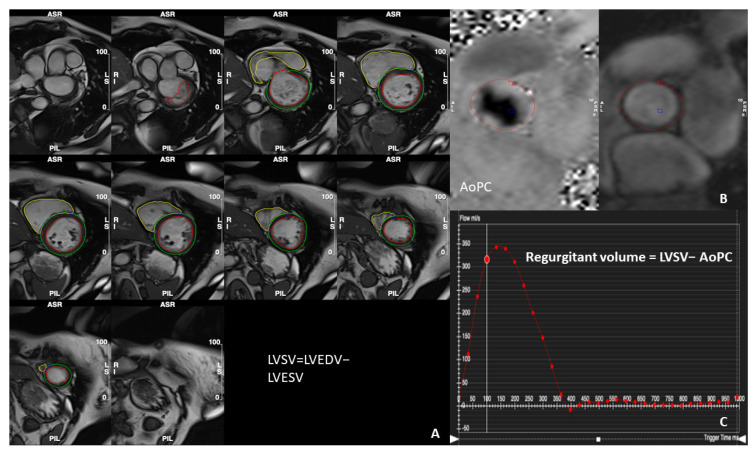
Standard quantification method of mitral regurgitation. (**A**). Examples of contours on diastolic frames of an SSFP short-axis cine stack. LV (red contours), RV (yellow contours). (**B**,**C**). Aortic valve through-plane flow image and flow graph. Mitral regurgitant volume is calculated by subtracting aortic forward flow from LVSV. LV, left ventricle; RV, right ventricle; LVSV, left ventricular stroke volume; LVEDV, left ventricular end diastolic volume; LVESV, left ventricular end-systolic volume; AoPC, aortic forward flow using phase contrast imaging.

CMR not only provides qualitative insights into MR, but also offers multiple methods for quantitative assessment, both direct and indirect (Table 1). The latter calculate the MR regurgitant volume (RVol) using flow measurements in other parts of the heart. Among these, the most common approach involves calculating the difference between planimetry-derived left ventricular (LV) stroke volume (SV) and the forward systolic volume measured using phase contrast (flow velocity encoded) mapping at the aortic root [19]. This method, which is shown in Figure 3, makes use of the robust short-axis (SA) cine stack analysis of the LV volumes and the highly reproducible phase contrast imaging at the level of the sinotubular junction in end-diastole, which has shown its accuracy in estimating forward and regurgitant blood flow through semiluminal (aortic and pulmonary) valves [9,20,21]. It does not need to account for the mitral regurgitant jet morphology, but it is still limited by the necessity for two separate acquisitions and the associated potential for error [22]. It can also be adapted to account for additional volume from concurrent aortic regurgitation, or in that same case, the phase contrast imaging plane could be set instead in the pulmonary artery, if no major intracardiac shunt exists [11]. The second “volumetric” method is the calculation of the difference between the LV SV and RV SV, both calculated using the slice summation technique using the same SA cine stack [19]. However, this is not applicable in cases with multiple valve lesions (e.g., tricuspid regurgitation, which is common especially in patients with secondary MR) or shunt flows and the inter- and intra-observer reproducibility of this technique has been shown to be relatively poor as RV contouring can be challenging and more prone to error in the SA stack images [19,22]. The third method that may be used involves quantifying the mitral inflow volume, and the aortic forward volume employing either 2D phase contrast imaging or the more advanced 4D flow analysis [23]. Given the substantial mobility of the mitral valve plane, the use of 2D phase contrast imaging can come with the cost of significant errors [22]. Many difficulties could also arise in those MR cases with eccentric jets, as accurately positioning the imaging plane perpendicular to the predominant flow direction, rather than the mitral valve plane itself, is crucial to avoid inaccurate measurements [24,25]. These challenges seem to be addressed using the promising 4D flow analysis, which is similar to classic phase contrast imaging but with flow velocity encoding in all three spatial directions and, additionally, that is relative to the dimension of time [26]. This technique could be advantageous as it could make possible, through specific post-processing, the accurate retrospective calculation of flow through any plane in the heart and major vessels with only single free-breathing, respiratory-navigated acquisition [26]. Four-dimensional flow CMR is highly reproducible and precise [27], as flows are quantified for the same averaged cardiac cycles, reducing errors due to heart rate variability and spatial misalignment. Theoretically, it is also suitable for the assessment of multiple valve lesions, and most shunt flows too.

Four-dimensional flow analysis can be employed to quantify MR RVol not only indirectly but also directly. This specifically involves the retrospective direct measurement of each dynamic regurgitant jet after pinpointing the mitral valve and adjusting a dynamic reconstructed plane so that it remains perpendicular to the jet throughout the regurgitation [28,29]. In the case of multiple jets a plane needs to be positioned appropriately for each individual jet, which can be a time-consuming process. However, data acceleration techniques such as radial under-sampling, generalized auto-calibrating partially parallel acquisitions (GRAPPA) and echo-planar imaging (EPI) have been developed, enabling a whole-heart 4D flow scan to be completed in approximately 10 min [30]. In that direction, semi- or fully automatic techniques enabling either flow tracking or valve tracking have been developed, which aid in this time-consuming process, with evidence pointing to the former technique as the most robust when compared to the volumetric indirect method of RVol quantification [31,32]. It is suitable for MR assessment also in concomitant valvular pathologies or shunt flows, but has been shown to be less reproducible in primary MR, mainly due to the complex and time-consuming plane reformatting process [27]. Based on a recent systematic review, more than 80% of recent studies have shown that the 4D flow analysis technique calculating mitral inflow and aortic forward flow for the MR RVol calculation exhibited the best inter- and intra-observer reproducibility [33]. All standard and novel methods for RVol quantification, summarized in the table below, can be used in routine practice for cross-validating, aiming for a comprehensive and robust assessment of the severity of MR.

## 3. Determination of the Severity of MR Using CMR Parameters

There is a shortage of data regarding specific thresholds for defining the severity of MR due to the absence of large trials with validated cohorts (Table 2). This is reflected in current international guidelines, which recommend identical cut-off limits for RVol and the regurgitant fraction (RF) in assessing MR both with echocardiography and CMR; although. recent studies and meta-analyses suggest that there could be a significant discrepancy between the two techniques particularly in patients with non-severe MR [7,8,34]. Heitner et al. and Penicka et al. have reported moderate agreement with the kappa coefficient being k = 0.47 and k = 0.48, respectively, whereas Jang et al. and Uretsky et al. have found this metric to be as low as k = 0.10 and k = 0.14, respectively, indicating a poor concordance between the two modalities [35,36,37,38]. In this direction, Gelfand et al. proposed that adopting RF = 42% as a cutoff value for severe MR aligns well with the Doppler echocardiography findings [39]. Interestingly, one study adopted a multiparametric approach using echocardiography as the reference and compared it to CMR RF [40]. The grading of MR severity exhibited excellent concordance and the authors proposed a CMR RF cutoff value of 35% to define significant MR [40].

In a group of patients that underwent mitral valve surgery, postoperative CMR and echocardiography, CMR showed superiority, reporting a substantial correlation between LV remodelling and MR severity (*p* < 0.0001) compared to echocardiography (*p* = 0.1) with the PISA method [50]. In a study conducted by Myerson et al., which followed initially asymptomatic patients with moderate or severe MR for up to 8 years, MR RVol and RF emerged as the most important MR metrics for determining the necessity of surgery [48]. The established threshold values were 55 mL for MR RVol and 40% for RF, with a progressively increasing risk associated with higher parameter values. The RF threshold for diagnosing severe MR in this study agrees with the one proposed by Polte et al. (RF > 41%) and is lower than the respective echocardiographic value for severity (RF ≥ 50%) [46]. Similarly, in the large prospective study by Penicka et al. with a follow-up of 258 asymptomatic patients with at least moderate MR over 5 years, RVol > 50 mL was found to have the highest accuracy predicting the combination of mortality and the indication for surgery, which is considerably lower than the 60 mL threshold proposed by the guidelines [36]. Given that RVol is directly related to LV size in primary MR, two patients with similar RVol values may exhibit varying degrees of MR, if their LV sizes differ. Calculating the RF helps overcome this issue by correcting RVol for LV size.

An additional advantage in the selection of asymptomatic patients requiring mitral valve correction may be offered with the assessment of extracellular volume (ECV). A recent prospective observational registry conducted by Kitkungvan et al. in patients with at least moderate primary MR demonstrated that RF and elevated ECV were independently associated with adverse events [51]. They identified a cutoff of 40% for RF and 30% for ECV as indicative of the need for mitral surgery.

Based on its accuracy in assessing LV volume and MR severity, and predicting LV reverse remodelling after correction, CMR should be employed not only to confirm the severity and help guide surgical decision-making but also to quantitatively assess the severity of MR in patients with equivocal findings on echocardiography. Importantly, additional large trials with validation cohorts are necessary to establish the optimal CMR cutoff values for MR severity.

## 4. Application of CMR in Primary and Secondary Mitral Regurgitation

With its multifaceted approach, CMR can offer significant value in the evaluation of MR regardless of its aetiology.

On one hand, CMR’s utility begins with the detailed valve apparatus assessment through high-resolution images that can depict the extent of rheumatic deformation or leaflet prolapse and the specific segments involved [52]. Concurrently, the accurate volumetric analysis of the LV is particularly beneficial to MVP patients as echocardiographic evaluation has been shown to potentially overestimate MR severity. Two reasons could contribute towards this overestimation. Firstly, echocardiography captures the regurgitation jet’s peak, when the coaptation defect of the leaflets is largest, which usually happens in mid–late systole. Therefore, assessing MR severity using echo-derived quantitative indices, like vena contracta or the flow convergence method, could be not only technically difficult due to the mainly eccentric nature of the jet, but also misleading as it extrapolates a measurement at the peak of the regurgitation to the whole duration of this phenomenon [53]. Instead, CMR is able to provide a more accurate calculation of the RVol using either the indirect or direct methods mentioned above. Secondly, Simpson’s biplane method could falsely underestimate LV end-systolic volume, neglecting the ventricular volume displaced into the left atrium, but contained within the prolapsed leaflets [54]. This issue can be addressed using CMR’s comprehensive 2D phase contrast or 4D flow analysis.

**Figure 4:**
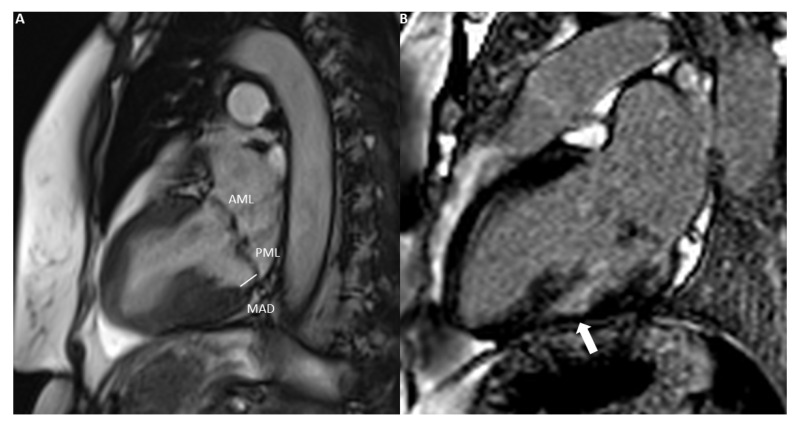
(**A**). Vertical long axis view demonstrating bilateral mitral valve prolapse with mitral annulus disjunction (detachment of the roots of the annulus from ventricular myocardium). (**B**). Predominantly subendocardial late gadolinium enhancement in mid-inferior wall (solid white arrow). Note also the LGE of the papillary muscle. Both MAD and presence of LGE are components of arrhythmic mitral valve prolapse. AML, anterior mitral leaflet; PML, posterior mitral leaflet; MAD, mitral annulus disjunction.

Another important contribution of CMR is its predictive value for MVP patients, as it can significantly aid in better risk stratification, detecting high-risk features such as bileaflet prolapse, extreme valve thickening, mitral annulus disjunction and systolic curling [55]. Interestingly, mitral annulus disjunction has been associated with sudden cardiac death due to ventricular arrhythmias in patients both with [14,16,56] and without evident MVP (Figure 4A) [15]. Transthoracic echocardiography has been found to have lower sensitivity in the detection of mitral annulus disjunction compared to CMR [16,17]. Beyond assessing morphology and function, CMR is unique in its ability to evaluate myocardial composition, particularly through its cornerstone technique, LGE imaging. It can detect fibrosis, often located in basal lateral or inferolateral segments or in papillary muscles, revealing important information that remains undetectable using other imaging modalities (Figure 4B) [12]. These areas of fibrosis constitute an integral part of LV remodelling and correlate well with both ventricular arrhythmias and different MR severity grades, with a markedly increased prevalence of LGE in moderate and severe MR patients [57]. There is also evidence to suggest that they are significantly associated with clinical outcomes, such as arrhythmias and sudden cardiac death, even after adjustment for the degree of MR severity and volume overload [45]. Decisions regarding Cardiac Resynchronization Therapy (CRT) can also be affected by fibrosis in these regions, as empirically CRT LV leads are usually placed in the posterior wall. This issue becomes even more pressing given that 30% of patients do not respond to CRT, especially those with ischaemic heart failure, thereby suggesting a central role of the ischaemic scar in the pathophysiology of this phenomenon [58]. Anatomical attributes, such as scar location and transmurality are important for the correct guidance of the lead placement, as if this fails it could lead to a lower response rate and up to a six-fold increase in mortality due to pump failure and malignant arrhythmias [59]. Additionally, there has been evidence of a correlation between worse clinical outcomes and persistent secondary MR after CRT, even after adjusting for LV reverse remodelling and especially if initially MR was moderate to severe [60]. This reveals that the decrease in secondary MR could play a distinct role in improving survival outcomes, beyond just reflecting volume changes. Therefore, CMR could be used not only for pinpointing the scar-free area that would have better chances for successful lead placement, but also for improving the patient selection process for possible structural intervention with percutaneous correction of persistent secondary MR.

With the additional use of feature tracking and parametric mapping, CMR could even pinpoint early myocardial tissue alterations, fibrosis, and the expansion of the interstitial space, surpassing the need of administering contrast [61]. T1 mapping has been shown to correlate with the extent of extracellular space and fibrosis, utilizing specialized modified Look-Locker pulse sequences over 9–17 heartbeats and generating the longitudinal magnetization inversion recovery curves [62]. With this process, different indices are calculated, which refer to the whole myocardial tissue, to each myocardial segment, and to each pixel location (hence the term mapping). The native (without contrast) T1 values increase, while interstitial fibrosis increases; post-contrast T1 values become shorter, and their combination in a formula that also uses the patient’s hematocrit allows for calculation of the ECV [63]. Various studies have suggested that both native T1 values and ECVs are increased in MR patients and have demonstrated significant prognostic value [51,64,65,66]. In addition to that, feature tracking involves retrospective processing of common SSFP images which tracks myocardium in a way that is similar to speckle tracking in advanced echocardiography [67]. It also enables the quantification of both global and regional indices for longitudinal, circumferential, and radial strain without the need for additional sequences and scanning time that the older strain analysis CMR tagging technique needed [68,69]. Therefore, it could detect alterations in myocardial tissue and function in the earlier stages of the disease process, before traditional metrics like ejection fraction start to decline. This capability was evident in a recent study by Guglielmo et. al., where asymptomatic MVP patients when compared with controls showed notably reduced global circumferential strain and regional circumferential and radial strain in the basal and mid inferolateral walls [70]. Native T1 values were also different in those regions, significantly higher in the MVP population, showing the multitude of information that we could extract from CMR even without gadolinium contrast [70]. Interestingly, these changes were not significantly correlated with the MR RVol, suggesting that myocardial alterations in MVP are a hallmark of a broader pathologic process where the stretching of the prolapsed leaflets is at least as important as chronic volume overload [70]. As a result, CMR can aid from a different, unique perspective towards a more thorough risk stratification for MVP patients.

On the other hand, CMR can be of value in the assessment of secondary mitral regurgitation as well. It can aid in the diagnostic work-up of various dilated cardiomyopathies, and it can also detect the extent of fibrosis and provide valuable information regarding revascularization and a concurrent mitral valve surgery in patients who are surgical candidates [71,72]. With its high spatial resolution and endocardial delineation, it can reliably assess LV remodelling and accurately quantify alterations in annular geometry, including septal–lateral and inter-commissural diameters, even when compared to the excellent resolution of transoesophageal echocardiography [73]. Such detailed assessments can guide surgical or percutaneous reparative approaches and monitor their outcome and long-term LV remodelling without the need for more invasive modalities and their associated risks [52]. This utility of CMR was highlighted in Hamilton-Craig et al.’s study, showcasing excellent reproducibility in patients predominantly with secondary MR that were treated invasively with percutaneous edge to edge repair [74]. In such scenarios, echocardiography may struggle with the multiple regurgitation jets and clip artifacts, presenting challenges in the accurate and consistent monitoring of MR, but CMR could be a promising alternative for a comprehensive follow-up. Beyond anatomical evaluation, which can illustrate LV dilatation and the resulting papillary muscle displacement and leaflet tethering, tissue characterization is also crucial. LGE imaging, specifically, can depict fibrosis within the papillary muscles or the LV myocardium offering insights into myocardial wall viability that are vital for decision making, as shown by Cavalcante et al. who suggested that the combination of RF > 35% and the extent of fibrosis in more than 30% of LV was detrimental for all-cause mortality or heart transplantation despite surgical intervention [44]. The extent of fibrosis has also been found to give valuable predictive information about the progression of ischaemic MR [72]. Additionally, previous studies have shown that papillary muscle LGE has a significant prognostic value in secondary MR patients. Firstly, in a study by Ivanov et al., although fibrosis in either papillary muscle was not correlated with adverse outcomes, the presence of scar in both papillary muscles was significantly associated with a higher risk of mortality and worsening heart failure [75]. Second, Flynn et al. showed that in patients undergoing coronary artery bypass graft surgery and concurrently mitral valve annuloplasty, the presence of extensive scarring in the posterior papillary muscle detected using a preoperative CMR was significantly associated with increased MR recurrence [50]. This suggests that screening for fibrosis in secondary MR patients could inform the choice between annuloplasty and valve replacement and potentially deem those patients ineligible for mitral annuloplasty, thus enhancing treatment outcomes. Last but not least, in a recent study shorter native T1 values in the pre-operative CMR in patients with functional MR were found to be of significant prognostic value for LV reverse remodelling six months post-surgery potentially indicating that higher T1 values could point towards a more fixed defect [76]. As a result, the authors suggested that monitoring native T1 values in asymptomatic patients with severe MR may aid in personalizing the timing of intervention.

## 5. Limitations of CMR in the Assessment of Patients with MR

While CMR offers a detailed and comprehensive approach to assessing MR patients, it is important to highlight its limitations.

Image quality heavily relies on patient’s heart rhythm. Arrhythmias, especially atrial fibrillation or frequent ectopic beats can potentially degrade image quality because CMR relies on ECG gating to synchronise data acquisition across multiple successive heart beats [77]. This averaging of images combined with the modality’s limitations in temporal resolution could make capturing the fast movements of mitral valve leaflets or associated fast-moving structures with variable positioning over the cardiac cycle (e.g., vegetations) challenging [78]. Furthermore, the limited spatial resolution of CMR, with most MR assessment protocols suggesting 5–6 mm slice thickness, hinders the detailed visualization of the direction of the mitral valve tip which usually has 1–5 mm thickness, thus making the differentiation between segment prolapse and flail less accurate [11,79,80]. Another factor that could potentially compromise CMR’s ability for precise MR assessment is the operator-dependent variability in cavity contouring. More specifically, even though CMR has been the reference standard for volume calculation, decisions regarding the inclusion or exclusion of papillary muscles and trabeculae could still significantly alter the measured volumes, consequently affecting RVol, RF, and, ultimately, the final assessment of MR severity [81,82]. Volume variability could also be affected by inconsistencies in including the basal slice in the slice summation technique, a problem exaggerated by the well-recognized through-plane motion of the mitral annulus [83]. Two-dimensional phase contrast imaging can also struggle with the continuously changing direction of the dynamic MR jets especially in MVP patients, making the case for the gradual necessity of 4D flow, which is unfortunately still not widely available [18]. Additionally, CMR itself is not universally available in contrast to echocardiography, requiring costly equipment and specialized expertise. The lack of standardized protocols among vendors especially in more advanced techniques such as parametric mapping, feature tracking, and 4D flow analysis, creates challenges in ensuring the reliability and robustness of the method across different institutions [63]. These factors together with the fact that there is still no gold-standard in the assessment of MR, highlight the need for a careful consideration of CMR’s position in the management pathway of MR patients.

## 6. Conclusions

CMR stands as a useful tool in the comprehensive evaluation of MR, as it offers detailed insights into both valvular and myocardial pathology. Although it provides unparalleled high-resolution volumetric assessment and tissue characterization imaging, its widespread application is limited by factors like cost, lack of standardization, and susceptibility to arrhythmia-related artifacts. As technology advances and new sequences or techniques like 4D flow become more clinically applicable, we expect to see a growing body of evidence correlating CMR indices with hard clinical outcomes in MR patients. This evolution will further cement CMR’s role in facilitating personalized and informed decision-making in cardiology.

## Figures and Tables

**Table 1:** Standard and novel CMR methods for quantification of mitral valve regurgitation. AoPC, aortic forward flow using phase contrast imaging; LV, left ventricle; LVSV, left ventricular stroke volume; LVEDV, left ventricular end diastolic volume; LVESV, left ventricular end-systolic volume; RV, right ventricle; RVSV, right ventricular stroke volume; VSD, ventricular septal defect.

Method		Strengths	Pitfalls
Standard method	Regurgitant volume = LVSV- AoPC (mL/cardiac cycle) Regurgitant fraction = (Regurgitant fraction/LV stroke volume) × 100%	Simple Highly reproducible Robust for almost all cardiac lesions (except for VSD) Applicable for eccentric or multiple jets	Depends on accurate flow data Uncontrolled arrythmia may reduce accuracy
Alternative method (Cine)	Regurgitant volume = LVSV − RVSV	Simple Can be used if flow imaging is unreliable/unavailable	Cannot be applied in presence of other lesions Less robust
4D flow direct tracking of mitral regurgitant jet		Direct quantification High reproducibility	Heterogenous correlations to conventional quantification methods Absence of a gold-standard Challenging in presence of multiple jets

**Table 2:** Thresholds used in bibliography to define severe mitral regurgitation. (i), indexed Regurgitant Volume; LAVi, indexed left atrial volume; LVEDVi, indexed left ventricular end diastolic volume; LVESVi, indexed left ventricular end-systolic volume.

		Thresholds for Severe Mitral Regurgitation				
Study	Population	Regurgitant Volume	Regurgitant Fraction	LVEDVi	LVESVi	LAVi
Uretsky et al., 2022 Ref. [41]	152 patients with degenerative MR	≥60 mL	≥50%	N/A	N/A	N/A
Uretsky et al., 2021 Ref. [42]	158 patients with primary MR and Presence of a flail leaflet or Coanda on echo	≥60 mL	≥50%	N/A	N/A	N/A
Capron et al., 2020 Ref. [43]	44 patients with moderate to severe chronic primary MR	≥60 mL	N/A	≥92 mL/m^2^	N/A	N/A
Cavalcante et al., 2020 Ref. [44]	578 patients with ICM and ischemic MR	N/A	≥35% (significant MR)	N/A	N/A	N/A
Kitkungvan et al., 2018 Ref. [45]	356 primary MR patients	N/A	≥50%	≥95 mL/m^2^	N/A	N/A
Penicka et al., 2018 Ref. [36]	258 asymptomatic patients with moderate/severe primary MR	≥60 mL	N/A	N/A	N/A	N/A
Polte et al., 2017 Ref. [46]	40 patients with moderate/severe MR on echo	>64 mL >32 mL/m^2^ (i)	>41%	120 mL/m^2^	N/A	N/A
Aplin et al., 2016 Ref. [47]	72 patients, primary MR on echocardiography	>39 mL >21 mL/m^2^ (i)	>27%	>108 mL/m^2^	>72 mL/m^2^	>83 mL/m^2^
Myerson et al., 2016 Ref. [48]	109, asymptomatic patients with moderate/severe MR on echo	>55 mL >29 mL/m^2^ (i)	>40%	≥95 mL/m^2^	>36 mL/m^2^	N/A
Uretsky et al., 2015 Ref. [49]	103 patients with MR on echocardiography	≥60 mL	N/A	N/A	N/A	N/A

## Data Availability

No new data were created.

## References

[1] Nkomo V.T., Gardin J.M., Skelton T.N., Gottdiener J.S., Scott C.G., Enriquez-Sarano M. (2006). Burden of valvular heart diseases: A population-based study. Lancet.

[2] Enriquez-Sarano M., Akins C.W., Vahanian A. (2009). Mitral regurgitation. Lancet.

[3] Iung B., Baron G., Butchart E.G., Delahaye F., Gohlke-Bärwolf C., Levang O.W., Tornos P., Vanoverschelde J.-L., Vermeer F., Boersma E. (2003). A prospective survey of patients with valvular heart disease in Europe: The Euro Heart Survey on Valvular Heart Disease. Eur. Heart J..

[4] El Sabbagh A., Reddy Y.N., Nishimura R.A. (2018). Mitral Valve Regurgitation in the Contemporary Era: Insights Into Diagnosis, Management, and Future Directions. JACC Cardiovasc. Imaging.

[5] Suinesiaputra A., Bluemke D.A., Cowan B.R., Friedrich M.G., Kramer C.M., Kwong R., Plein S., Schulz-Menger J., Westenberg J.J.M., Young A.A. (2015). Quantification of LV function and mass by cardiovascular magnetic resonance: Multi-center variability and consensus contours. J. Cardiovasc. Magn. Reson..

[6] Uretsky S., Argulian E., Narula J., Wolff S.D. (2018). Use of Cardiac Magnetic Resonance Imaging in Assessing Mitral Regurgitation: Current Evidence. J. Am. Coll. Cardiol..

[7] Otto C.M., Nishimura R.A., Bonow R.O., Carabello B.A., Erwin J.P., Gentile F., Jneid H., Krieger E.V., Mack M., Writing Committee Members (2021). 2020 ACC/AHA Guideline for the Management of Patients With Valvular Heart Disease: Executive Summary: A Report of the American College of Cardiology/American Heart Association Joint Committee on Clinical Practice Guidelines. J. Am. College Cardiol..

[8] Vahanian A., Beyersdorf F., Praz F., Milojevic M., Baldus S., Bauersachs J., Capodanno D., Conradi L., De Bonis M., De Paulis R. (2021). 2021 ESC/EACTS Guidelines for the management of valvular heart disease. Eur. Heart J..

[9] Kramer C.M., Barkhausen J., Bucciarelli-Ducci C., Flamm S.D., Kim R.J., Nagel E. (2020). Standardized cardiovascular magnetic resonance imaging (CMR) protocols: 2020 update. J. Cardiovasc. Magn. Reson..

[10] Lopez-Mattei J.C., Shah D.J. (2013). The Role of Cardiac Magnetic Resonance in Valvular Heart Disease. Methodist DeBakey Cardiovasc. J..

[11] Garg P., Swift A.J., Zhong L., Carlhäll C.-J., Ebbers T., Westenberg J., Hope M.D., Bucciarelli-Ducci C., Bax J.J., Myerson S.G. (2020). Assessment of mitral valve regurgitation by cardiovascular magnetic resonance imaging. Nat. Rev. Cardiol..

[12] Han Y., Peters D.C., Salton C.J., Bzymek D., Nezafat R., Goddu B., Kissinger K.V., Zimetbaum P.J., Manning W.J., Yeon S.B. (2008). Cardiovascular Magnetic Resonance Characterization of Mitral Valve Prolapse. JACC Cardiovasc. Imaging.

[13] Sturla F., Onorati F., Puppini G., Pappalardo O.A., Selmi M., Votta E., Faggian G., Redaelli A. (2017). Dynamic and quantitative evaluation of degenerative mitral valve disease: A dedicated framework based on cardiac magnetic resonance imaging. J. Thorac. Dis..

[14] Marra M.P., Basso C., De Lazzari M., Rizzo S., Cipriani A., Giorgi B., Lacognata C., Rigato I., Migliore F., Pilichou K. (2016). Morphofunctional Abnormalities of Mitral Annulus and Arrhythmic Mitral Valve Prolapse. Circ. Cardiovasc. Imaging.

[15] Dejgaard L.A., Skjølsvik E.T., Lie Ø.H., Ribe M., Stokke M.K., Hegbom F., Scheirlynck E.S., Gjertsen E., Andresen K., Helle-Valle T.M. (2018). The Mitral Annulus Disjunction Arrhythmic Syndrome. J. Am. Coll. Cardiol..

[16] Essayagh B., Iacuzio L., Civaia F., Avierinos J.-F., Tribouilloy C., Levy F. (2019). Usefulness of 3-Tesla Cardiac Magnetic Resonance to Detect Mitral Annular Disjunction in Patients With Mitral Valve Prolapse. Am. J. Cardiol..

[17] Mantegazza V., Volpato V., Gripari P., Ali S.G., Fusini L., Italiano G., Muratori M., Pontone G., Tamborini G., Pepi M. (2021). Multimodality imaging assessment of mitral annular disjunction in mitral valve prolapse. Heart.

[18] Vermes E., Iacuzio L., Levy F., Bohbot Y., Renard C., Gerber B., Maréchaux S., Tribouilloy C. (2022). Role of Cardiovascular Magnetic Resonance in Native Valvular Regurgitation: A Comprehensive Review of Protocols, Grading of Severity, and Prediction of Valve Surgery. Front. Cardiovasc. Med..

[19] Polte C.L., Bech-Hanssen O., Johnsson A., Gao S.A., Lagerstrand K.M. (2015). Mitral regurgitation quantification by cardiovascular magnetic resonance: A comparison of indirect quantification methods. Int. J. Cardiovasc. Imaging.

[20] Hundley W.G., Li H.F., Hillis L.D., Meshack B.M., Lange R.A., Willard J.E., Landau C., Peshock R.M. (1995). Quantitation of cardiac output with velocity-encoded, phase-difference magnetic resonance imaging. Am. J. Cardiol..

[21] Zoghbi W.A., Adams D., Bonow R.O., Enriquez-Sarano M., Foster E., Grayburn P.A., Hahn R.T., Han Y., Hung J., Lang R.M. (2017). Recommendations for Noninvasive Evaluation of Native Valvular Regurgitation. J. Am. Soc. Echocardiogr..

[22] Kon M.W., Myerson S.G., Moat N.E., Pennell D.J. (2004). Quantification of regurgitant fraction in mitral regurgitation by cardio-vascular magnetic resonance: Comparison of techniques. J. Heart Valve Dis..

[23] Gatehouse P.D., Rolf M.P., Graves M.J., Hofman M.B., Totman J., Werner B., Quest R.A., Liu Y., von Spiczak J., Dieringer M. (2010). Flow measurement by cardiovascular magnetic resonance: A multi-centre multi-vendor study of background phase offset errors that can compromise the accuracy of derived regurgitant or shunt flow measurements. J. Cardiovasc. Magn. Reson..

[24] Kilner P.J., Gatehouse P.D., Firmin D.N. (2007). Flow measurement by magnetic resonance: A unique asset worth optimising. J. Cardiovasc. Magn. Reson..

[25] Myerson S.G., Francis J.M., Neubauer S. (2010). Direct and indirect quantification of mitral regurgitation with cardiovascular magnetic resonance, and the effect of heart rate variability. Magn. Reson. Mater. Phys. Biol. Med..

[26] Dyverfeldt P., Bissell M., Barker A.J., Bolger A.F., Carlhäll C.-J., Ebbers T., Francios C.J., Frydrychowicz A., Geiger J., Giese D. (2015). 4D flow cardiovascular magnetic resonance consensus statement. J. Cardiovasc. Magn. Reson..

[27] Fidock B., Archer G., Barker N., Elhawaz A., Al-Mohammad A., Rothman A., Hose R., Hall I.R., Grech E., Briffa N. (2021). Standard and emerging CMR methods for mitral regurgitation quantification. Int. J. Cardiol..

[28] Lee J., Gupta A.N., Ma L.E., Scott M.B., Mason O.R., Wu E., Thomas J.D., Markl M. (2022). Valvular regurgitation flow jet assessment using in vitro 4D flow MRI: Implication for mitral regurgitation. Magn. Reson. Med..

[29] Feneis J.F., Kyubwa E., Atianzar K., Cheng J.Y., Alley M.T., Vasanawala S.S., Demaria A.N., Hsiao A. (2018). 4D flow MRI quantification of mitral and tricuspid regurgitation: Reproducibility and consistency relative to conventional MRI. J. Magn. Reson. Imaging.

[30] Garg P., Westenberg J.J., Bsc P.J.V.D.B., Swoboda P.P., Aziz R., Foley J.R., Fent G.J., Tyl F., Coratella L., ElBaz M.S. (2018). Comparison of fast acquisition strategies in whole-heart four-dimensional flow cardiac MR: Two-center, 1.5 Tesla, phantom and in vivo validation study. J. Magn. Reson. Imaging.

[31] Ma L.E., Yerly J., Piccini D., Di Sopra L., Roy C.W., Carr J.C., Rigsby C.K., Kim D., Stuber M., Markl M. (2020). 5d flow mri: A fully self-gated, free-running framework for cardiac and respiratory motion–resolved 3d hemodynamics. Radiol. Cardiothorac. Imaging.

[32] Blanken C.P.S., Westenberg J.J.M., Aben J.-P., Bijvoet G.P., Chamuleau S.A.J., Boekholdt S.M., Nederveen A.J., Leiner T., Van Ooij P., Planken R.N. (2020). Quantification of mitral valve regurgitation from 4D flow MRI using semiautomated flow tracking. Radiol. Cardiothorac. Imaging.

[33] Safarkhanlo Y., Jung B., Bernhard B., Peper E.S., Kwong R.Y., Bastiaansen J.A.M., Gräni C. (2023). Mitral valve regurgitation assessed by intraventricular CMR 4D-flow: A systematic review on the technological aspects and potential clinical applications. Int. J. Cardiovasc. Imaging.

[34] Botis I., Efstathiadou A., Papanastasiou C.A., Kokkinidis D.G., Zegkos T., Efthimiadis G., Kamperidis V., Khalique O.K., Kampaktsis P.N., Karamitsos T.D. (2021). Evaluation of mitral regurgitation by cardiac magnetic resonance and transthoracic echocardiography: A systematic review and meta-analysis. Rev. Cardiovasc. Med..

[35] Heitner J., Bhumireddy G.P., Crowley A.L., Weinsaft J., Haq S.A., Klem I., Kim R.J., Jollis J.G. (2012). Clinical application of cine-MRI in the visual assessment of mitral regurgitation compared to echocardiography and cardiac catheterization. PLoS ONE.

[36] Penicka M., Vecera J., Mirica D.C., Kotrc M., Kockova R., Van Camp G. (2018). Prognostic Implications of Magnetic Resonance–Derived Quantification in Asymptomatic Patients With Organic Mitral Regurgitation: Comparison with Doppler Echocardiography-Derived Integrative Approach. Circulation.

[37] Jang J.Y., Kang J.-W., Yang D.H., Lee S., Sun B.J., Kim D.-H., Song J.-M., Kang D.-H., Song J.-K. (2018). Impact of a Geometric Correction for Proximal Flow Constraint on the Assessment of Mitral Regurgitation Severity Using the Proximal Flow Convergence Method. J. Cardiovasc. Ultrasound.

[38] Uretsky S., Argulian E., Marcoff L., Koulogiannis K., Supariwala A., Rosenthal M., Brown M.J., Jagarlamudi A., Chaudhry F., Awan H. (2015). A Comparative Assessment of Echocardiographic Parameters for Determining Mitral Re-gurgitation Severity. Circulation.

[39] Gelfand E.V., Hughes S., Hauser T.H., Yeon S.B., Goepfert L., Kissinger K.V., Rofsky N.M., Manning W.J. (2006). Severity of mitral and aortic regurgitation as assessed by cardiovascular magnetic resonance: Optimizing correlation with Doppler echocardiography. J. Cardiovasc. Magn. Reson..

[40] Le Goffic C., Toledano M., Ennezat P.-V., Binda C., Castel A.-L., Delelis F., Graux P., Tribouilloy C., Maréchaux S. (2015). Quantitative Evaluation of Mitral Regurgitation Secondary to Mitral Valve Prolapse by Magnetic Resonance Imaging and Echocardiography. Am. J. Cardiol..

[41] Uretsky S., Animashaun I.B., Sakul S., Aldaia L., Marcoff L., Koulogiannis K., Argulian E., Rosenthal M., Wolff S.D., Gillam L.D. (2022). American Society of Echocardiography Algorithm for Degenerative Mitral Regurgitation: Comparison With CMR. JACC Cardiovasc. Imaging.

[42] Uretsky S., Morales D.C.V., Aldaia L., Mediratta A., Koulogiannis K., Marcoff L., Sakul S., Wolff S.D., Gillam L.D. (2021). Characterization of Primary Mitral Regurgitation With Flail Leaflet and/or Wall-Impinging Flow. J. Am. Coll. Cardiol..

[43] Capron T., Cautela J., Scemama U., Miola C., Bartoli A., Theron A., Pinto J., Porto A., Collart F., Lepidi H. (2020). Cardiac magnetic resonance assessment of left ventricular dilatation in chronic severe left-sided regurgitations: Comparison with standard echocardiography. Diagn. Interv. Imaging.

[44] Cavalcante J.L., Kusunose K., Obuchowski N.A., Jellis C., Griffin B.P., Flamm S.D., Kwon D.H. (2020). Prognostic Impact of Ischemic Mitral Regurgitation Severity and Myocardial Infarct Quantification by Cardiovascular Magnetic Resonance. JACC Cardiovasc. Imaging.

[45] Kitkungvan D., Nabi F., Kim R.J., Bonow R.O., Khan M.A., Xu J., Little S.H., Quinones M.A., Lawrie G.M., Zoghbi W.A. (2018). Myocardial Fibrosis in Patients With Primary Mitral Regurgitation With and Without Prolapse. J. Am. Coll. Cardiol..

[46] Polte C.L., Gao S.A., Johnsson Å.A., Lagerstrand K.M., Bech-Hanssen O. (2017). Characterization of Chronic Aortic and Mitral Regurgitation Undergoing Valve Surgery Using Cardiovascular Magnetic Resonance. Am. J. Cardiol..

[47] Aplin M., Kyhl K., Bjerre J., Ihlemann N., Greenwood J.P., Plein S., Uddin A., Tønder N., Høst N.B., Ahlström M.G. (2016). Cardiac remodelling and function with primary mitral valve insufficiency studied by magnetic resonance imaging. Eur. Heart J. Cardiovasc. Imaging.

[48] Myerson S.G., d’Arcy J., Christiansen J.P., Dobson L.E., Mohiaddin R., Francis J.M., Prendergast B., Greenwood J.P., Karamitsos T.D., Neubauer S. (2016). Determination of Clinical Outcome in Mitral Regurgitation With Cardiovascular Magnetic Resonance Quantification. Circulation.

[49] Uretsky S., Gillam L., Lang R., Chaudhry F.A., Argulian E., Supariwala A., Gurram S., Jain K., Subero M., Jang J.J. (2015). Discordance between echocardiography and MRI in the assessment of mitral regurgitation severity: A prospective multicenter trial. J. Am. Coll. Cardiol..

[50] Flynn M., Curtin R., Nowicki E.R., Rajeswaran J., Flamm S.D., Blackstone E.H., Mihaljevic T. (2009). Regional wall motion abnormalities and scarring in severe functional ischemic mitral regurgitation: A pilot cardiovascular magnetic resonance imaging study. J. Thorac. Cardiovasc. Surg..

[51] Kitkungvan D., Yang E.Y., El Tallawi K.C., Nagueh S.F., Nabi F., Khan M.A., Nguyen D.T., Graviss E.A., Lawrie G.M., Zoghbi W.A. (2019). Prognostic Implications of Diffuse Interstitial Fibrosis in Asymptomatic Primary Mitral Regurgitation. Circulation.

[52] Mehta N.K., Kim J., Siden J.Y., Rodriguez-Diego S., Alakbarli J., Di Franco A., Weinsaft J.W. (2017). Utility of cardiac magnetic resonance for evaluation of mitral regurgitation prior to mitral valve surgery. J. Thorac. Dis..

[53] Topilsky Y., Michelena H., Bichara V., Maalouf J., Mahoney D.W., Enriquez-Sarano M. (2012). Mitral valve prolapse with mid-late systolic mitral regurgitation: Pitfalls of evaluation and clinical outcome compared with holosystolic regurgitation. Circulation.

[54] Pavon A.G., Guglielmo M., Mennilli P.M., Falcão M.B.L., Bergamaschi L., Costantin D.F., Vivaldo M., Leo L.A., Schlossbauer S., Roy C.W. (2022). The Role of Cardiovascular Magnetic Resonance in Patients with Mitral Regurgitation. J. Cardiovasc. Dev. Dis..

[55] Pavon A.G., Monney P., Schwitter J. (2021). Mitral Valve Prolapse, Arrhythmias, and Sudden Cardiac Death: The Role of Multimodality Imaging to Detect High-Risk Features. Diagnostics.

[56] Basso C., Marra M.P., Rizzo S., De Lazzari M., Giorgi B., Cipriani A., Frigo A.C., Rigato I., Migliore F., Pilichou K. (2015). Arrhythmic Mitral Valve Prolapse and Sudden Cardiac Death. Circulation.

[57] Beaufils A.-L.C.D., Huttin O., Jobbe-Duval A., Senage T., Filippetti L., Piriou N., Cueff C., Venner C., Mandry D., Sellal J.-M. (2021). Replacement Myocardial Fibrosis in Patients With Mitral Valve Prolapse. Circulation.

[58] Auricchio A., Prinzen F.W. (2011). Non-responders to cardiac resynchronization therapy: The magnitude of the problem and the issues. Circ. J..

[59] Leyva F., Foley P.W., Chalil S., Ratib K., Smith R.E., Prinzen F., Auricchio A. (2011). Cardiac resynchronization therapy guided by late gadolinium-enhancement cardiovascular magnetic resonance. J. Cardiovasc. Magn. Reson..

[60] van der Bijl P., Khidir M., Marsan N.A., Delgado V., Leon M.B., Stone G.W., Bax J.J. (2019). Effect of Functional Mitral Regurgitation on Outcome in Patients Receiving Cardiac Resynchronization Therapy for Heart Failure. Am. J. Cardiol..

[61] Bui A.H., Roujol S., Foppa M., Kissinger K.V., Goddu B., Hauser T.H., Zimetbaum P.J., Ngo L.H., Manning W.J., Nezafat R. (2017). Diffuse myocardial fibrosis in patients with mitral valve prolapse and ventricular arrhythmia. Heart.

[62] Taylor A.J., Salerno M., Dharmakumar R., Jerosch-Herold M. (2016). T1 Mapping: Basic Techniques and Clinical Applications. JACC Cardiovasc. Imaging.

[63] Podlesnikar T., Delgado V., Bax J.J. (2018). Cardiovascular magnetic resonance imaging to assess myocardial fibrosis in valvular heart disease. Int. J. Cardiovasc. Imaging.

[64] Miller C.A., Naish J.H., Bishop P., Coutts G., Clark D., Zhao S., Ray S.G., Yonan N., Williams S.G., Flett A.S. (2013). Schmitt, Comprehensive validation of cardiovascular magnetic resonance techniques for the as-sessment of myocardial extracellular volume. Circ. Cardiovasc. Imaging.

[65] Badau C.I.R., Coldea L.A. (2022). The value of T1 mapping in patients with chronic mitral regurgitation. Eur. Heart J..

[66] Agbor-Etang B.B., Lim L.J., Ordovas K.G., Delling F.N. (2020). Evidence of Abnormal T1 Mapping in Arrhythmic Mitral Valve Prolapse Without Significant Mitral Regurgitation. Circulation.

[67] Hor K.N., Baumann R., Pedrizzetti G., Tonti G., Gottliebson W.M., Taylor M., Benson W., Mazur W. (2011). Magnetic Resonance Derived Myocardial Strain Assessment Using Feature Tracking. J. Vis. Exp..

[68] Zerhouni E.A., Parish D.M., Rogers W.J., Yang A., Shapiro E.P. (1988). Human heart: Tagging with MR imaging--a method for noninvasive assessment of myocardial motion. Radiology.

[69] Schuster A., Hor K.N., Kowallick J.T., Beerbaum P., Kutty S. (2016). Cardiovascular Magnetic Resonance Myocardial Feature Tracking. Circ. Cardiovasc. Imaging.

[70] Guglielmo M., Fusini L., Muscogiuri G., Baessato F., Loffreno A., Cavaliere A., Rizzon G., Baggiano A., Rabbat M.G., Muratori M. (2021). T1 mapping and cardiac magnetic resonance feature tracking in mitral valve prolapse. Eur. Radiol..

[71] Arbelo E., Protonotarios A., Gimeno J.R., Arbustini E., Barriales-Villa R., Basso C., Bezzina C.R., Biagini E., Blom N.A., de Boer R.A. (2023). 2023 ESC Guidelines for the management of cardiomyopathies: Developed by the task force on the management of cardiomyopathies of the European So-ciety of Cardiology (ESC). Eur. Heart J..

[72] Kwon D.H., Kusunose K., Obuchowski N.A., Cavalcante J.L., Popovic Z.B., Thomas J.D., Desai M.Y., Flamm S.D., Griffin B.P. (2016). Predictors and Prognostic Impact of Progressive Ischemic Mitral Regurgitation in Patients With Advanced Ischemic Cardiomyopathy: A Multimodality Study. Circ. Cardiovasc. Imaging.

[73] Kaji S., Nasu M., Yamamuro A., Tanabe K., Nagai K., Tani T., Tamita K., Shiratori K., Kinoshita M., Senda M. (2005). Annular geometry in patients with chronic ischemic mitral regurgitation: Three-dimensional magnetic resonance imaging study. Circulation.

[74] Hamilton-Craig C., Strugnell W., Gaikwad N., Ischenko M., Speranza V., Chan J., Neill J., Platts D., Scalia G.M., Burstow D.J. (2015). Quantitation of mitral regurgitation after percutaneous MitraClip repair: Comparison of Doppler echocardiography and cardiac magnetic resonance imaging. Ann. Cardiothorac. Surg..

[75] Ivanov A., Bhumireddy G., Dabiesingh D., Khan S., Ho J., Krishna N., Dontineni N., Socolow J., Briggs W., Klem I. (2016). Importance of papillary muscle infarction detected by cardiac magnetic resonance imaging in predicting cardiovascular events. Int. J. Cardiol..

[76] von Stumm M., Petersen J., Sinn M., Holst T., Sequeira-Gross T.M., Müller L., Pausch J., Bannas P., Adam G., Reichenspurner H. (2023). Correlation of Myocardial Native T1 and Left Ventricular Reverse Remodeling after Valvular Surgery. J. Clin. Med..

[77] Mathew R.C., Löffler A.I., Salerno M. (2018). Role of Cardiac Magnetic Resonance Imaging in Valvular Heart Disease: Diagnosis, Assessment, and Management. Curr. Cardiol. Rep..

[78] Myerson S.G. (2012). Heart valve disease: Investigation by cardiovascular magnetic resonance. J. Cardiovasc. Magn. Reson..

[79] Gabriel R.S., Kerr A.J., Raffel O.C., Stewart R.A., Cowan B.R., Occleshaw C.J. (2008). Mapping of mitral regurgitant defects by cardiovascular magnetic resonance in moderate or severe mitral regurgitation secondary to mitral valve prolapse. J. Cardiovasc. Magn. Reson..

[80] Søgaard S.B., Gustavsen P., Dalsgaard M., Vejlstrup N.G., Madsen P.L. (2021). Cardiac magnetic resonance imaging with standard imaging planes for mitral valve scallop pathology: Interrater agreement and comparison with echocardiography. Int. J. Cardiovasc. Imaging.

[81] Chuang M.L., Gona P., Hautvast G.L., Salton C.J., Blease S.J., Yeon S.B., Breeuwer M., O’Donnell C.J., Manning W.J. (2012). Correlation of Trabeculae and Papillary Muscles With Clinical and Cardiac Characteristics and Impact on CMR Measures of LV Anatomy and Function. JACC Cardiovasc. Imaging.

[82] Papavassiliu T., Kühl H.P., Schröder M., Süselbeck T., Bondarenko O., Böhm C.K., Beek A., Hofman M.M.B., van Rossum A.C. (2005). Effect of Endocardial Trabeculae on Left Ventricular Measurements and Measurement Reproducibility at Cardiovascular MR Imaging. Radiology.

[83] Marcus J.T., Götte M.J.W., Dewaal L.K., Stam M.R., Van der Geest R.J., Heethaar R.M., Van Rossum A.C. (1999). The influence of through-plane motion on left ventricular volumes measured by magnetic resonance imaging: Implications for image acquisition and analysis. J. Cardiovasc. Magn. Reson..

